# Renal resistive index in chronic kidney disease patients: Possible determinants and risk profile

**DOI:** 10.1371/journal.pone.0230020

**Published:** 2020-04-01

**Authors:** Michele Provenzano, Laura Rivoli, Carlo Garofalo, Teresa Faga, Elena Pelagi, Maria Perticone, Raffaele Serra, Ashour Michael, Nicolino Comi, Michele Andreucci

**Affiliations:** 1 Nephrology Unit of Magna Graecia University in Catanzaro, Catanzaro, Italy; 2 Division of Nephrology, Department of Scienze Mediche e Chirurgiche Avanzate, University of Campania “Luigi Vanvitelli”, Naples, Italy; 3 Department of Experimental and Clinical Medicine, Magna Græcia University, Catanzaro, Italy; 4 Interuniversity Center of Phlebolymphology (CIFL), International Research and Educational Program in Clinical and Experimental Biotechnology, Headquarters: Magna Graecia University of Catanzaro, Catanzaro, Italy; International University of Health and Welfare, School of Medicine, JAPAN

## Abstract

**Background:**

High ultrasound renal resistive index (RI) predicts poor cardiorenal outcomes in chronic kidney disease (CKD) and has recently emerged as a marker of nephroprotective drugs response. Thus, having a risk profile of CKD patients with abnormal RI may be relevant for the clinicians.

**Methods:**

Consecutive patients referred to our non-dialysis CKD clinic from 01/01/2016 to 01/12/2016, were evaluated by clinical and ultrasound analysis. Inclusion criteria were age >18 years and presence of CKD defined as estimated glomerular filtration rate (eGFR)<60 mL/min/1.73m^2^ and/or proteinuria>0.150g/24h. Renal artery stenosis, solitary kidney, acute kidney injury were the main exclusion criteria. RI value was the mean of three measures in segmental arteries in each kidney. Univariate analysis was performed to evaluate associations between continuous RI and clinical variables. Multivariate linear regression analysis, based on stepwise method with an elimination criterion of *p*<0.10, was used to assess the independent correlates of RI as continuous variable.

**Results:**

We studied 73 patients (69.9% men). Mean RI was 0.67±0.09. Frequencies of diabetes and cardiovascular disease (CVD) were 19.2% and 20.6% and median eGFR 54.1 [30.0–84.6] mL/min/1.73m^2^. From low (<0.65) to intermediate (0.65–0.70) to high (>0.70) RI categories, eGFR and haemoglobin levels were decreased while diabetes, cardiovascular disease (CVD), phosphate and smokers were higher. At univariate analysis, RI was significantly associated with age, presence of diabetes, CVD, serum phosphorus, eGFR, Urea and haemoglobin. Multi-adjusted stepwise regression analysis showed that lower eGFR levels (p<0.001), diabetes (p = 0.042), CVD (p = 0.009), smoking habit (p = 0.021) and higher serum phosphorus levels (p = 0.001) were associated with higher continuous RI. Serum phosphorus showed Area Under the Curves (AUC) values of 0.714 and 0.664 for discriminating RI cut-offs of 0.70 and 0.65.

**Conclusions:**

This analysis suggests that RI is higher in CKD patients with CVD, diabetes, smoking habit and higher serum phosphorus, regardless of eGFR. Further studies are needed to verify whether higher RI indicates more complex pathway of intrarenal damage, besides and beyond kidney function.

## Background

Optimizing risk stratification of chronic kidney disease (CKD) patients referred to tertiary nephrology care is a central issue of current Nephrology research because identification of high-risk patients allows nephrologists to better focus on patients who require closer monitoring and treatment to improve outcome as well as timely planning of renal substitutive therapy [1–6]. In the past few years there has been a growing attention to markers of subclinical renal damage, because they provide an accurate prediction of global cardio-renal outcome. Therefore, different parameters determined by using various imaging procedures have been introduced to improve the assessment of CKD severity. The primary preferred imaging procedure in the assessment of kidney diseases is ultrasonography (US), because it is inexpensive, noninvasive and easy to access [7]. It has been demonstrated, indeed, that doppler ultrasonographic renal resistive index (RI) shows a positive association with tubulo-interstitial and vascular lesions, and thus a well established inverse association with the eGFR levels, that is, the higher RI is, the higher the degree of kidney damage [8,9]. More important, RI was identified as a significant predictor of cardiovascular and renal outcomes in a wide setting of patients, regardless of eGFR and urine protein levels [10–12]. These important studies answer the question whether ultrasound parameters associate with eGFR decline and thus predict CKD progression or cardiovascular outcome, but not the other important clinical question namely whether doppler RI *per se* is associated with many other important risk factors linked to the clinical corollary of CKD, such as age, gender, presence of previous cardiovascular disease or diabetes and other metabolic assessments, regardless of eGFR. This concept, which can be defined as a risk profile of patients with higher RI levels, could help nephrologists to decide whether to schedule a US assessment, in addition to routine outpatient visit, among patients with CKD. Moreover, a recent pilot study has reinforced the importance of RI, by demonstrating that acute treatment with the sodium-glucose-cotransporter-2 inhibitor Dapagliflozin, in patients with type II diabetes, improves systemic endothelial function and RI as well [13]. A similar effect has been shown by RAAS-inhibitors, which have demonstrated the capacity to reduce RI, by reducing renal plasma flow through the vasodilation of the efferent arteriole [14] This new perspective of RI, as a marker of drug-response, represents a further reason to know what are the main risk factors associated with raised RI levels, in order to select patients that could benefit from a new treatment or be included in future clinical studies. We, thus, investigated the determinants of RI in a cohort of patients referred to tertiary nephrology care.

## Methods

### Study design and procedures

This is a cross-sectional clinical study examining 73 consecutive patients referred to our non-dialysis CKD clinic from January 1^st^, to December 1^st^, 2016. The cohort was originally built to collect information about the role of ultrasound parameters on the cardiovascular and renal risk stratification of patients referred to tertiary nephrology care. The study was approved by the Local Ethical Committee i.e. Calabria Region–Area Center Section and all patients gave written informed consent. Inclusion criteria were patients with age > 18 years, presence of CKD defined as: eGFR <60 mL/min/1.73 m^2^ and/or proteinuria > 0.150 g/24h for at least 3 months. Patients with renal artery stenosis, acute kidney injury, obstructive nephropathy, life expectancy <6 months, advanced liver or heart disease, solitary kidney and congenital abnormalities were excluded. Additional exclusion criteria were a history of renal replacement therapy, such as dialysis or kidney transplantation. Ultrasonographic studies were carried out by a 4.0-MHz curvilinear probe and a LOGIQ C5 Premium ultrasound machine (GE Healthcare, Zipf, Austria) using standard duplex Doppler sonography. US was performed by a nephrologist with at least a 10-years’ experience in renal US and who was blinded to patient history and laboratory results. To reduce the intraobserver variability, each measurement was repeated twice in the same session, and the average values were taken into account. RI was calculated as [(peak-systolic velocity − end-diastolic velocity)/peak-systolic velocity], on 3 segmental arteries (superior, middle, and inferior) in each kidney. The values were then averaged to obtain the mean value for each participant. In the same morning as the US study, nephrologists collected the medical history including CKD primitive diagnoses, previous cardiovascular disease (CVD: stroke, coronary heart disease, heart failure, peripheral vascular disease) and smoking habit, performed physical examination and registered laboratory results, therapy and events in anonymous electronic case reports. CKD primitive diagnoses were grouped as diabetic nephropathy (DN), hypertensive nephropathy (HTN), glomerulonephritis (GN), tubulo-intersitial nephritis (TIN) or polycystic kidney disease (PKD). Diagnosis of GN was biopsy-proven for all patients. GFR was estimated by the Chronic Kidney Disease Epidemiology Collaboration equation. Clinical and laboratory assessments were recorded at basal visit only and not repeated over time. The primary aim of this study was to search for the main determinants of RI modeled as continuous variable. As secondary analysis, diagnostic performances of the main RI determinants on the two reference RI thresholds most used in clinical practice, 0.65 and 0.70 [10,15,16], have been evaluated.

### Statistical analysis

Continuous variables were reported as either mean ± standard deviation (SD) or median and interquartile range (IQR) based on their distribution. Comparison among RI risk categories was assessed by one-way ANOVA or Kruskall-Wallis test. Categorical variables were analyzed using Chi-square test. For descriptive purposes, clinical and demographic variables were shown by RI categories with cut-offs at 0.65 and 0.70, respectively. For the model building process, univariate analysis testing the association between the main clinical variables and RI, modeled as continuous variable was assessed by means of linear regression analysis. The variables with p<0.15 at univariate analysis were selected and included in the first multivariate regression model (Model 1). Next, backward variable selection method with an elimination criterion of *p*<0.10 was performed to fit the second multivariate linear regression model (Model 2). Such as stringent cut-off for variables inclusion was used in order to avoid model overfitting, due to the limited sample of the cohort. Multicollinearity was assessed with variance inflation factors (VIF), which is a measure of the degree to which a single predictor variable can be expressed as a linear combination of the remaining predictor variables; values greater than 10 were cause for concern [17]. The final model was adjusted by: type-II diabetes, CVD, eGFR, serum phosphorus levels, and smoking habit. In the multivariate analysis (Model 2), the contribution of each covariate to the model fit was estimated as percentage reduction of R^2^ value of the model resulting from omitting each variable in turn from the full model [18]. We calculated R^2^s according to Nagelkerke [19]. First order interactions effects between covariates for the change in RI were also tested from the final model (Model 2). The receiver operating characteristic (ROC) curves were used to evaluate the variable’s ability for classifying disease status, which is, higher versus lower RI. RI cut-offs used to construct the ROC curves were 0.65 and 0.70, respectively. Comparison between ROC curves was assessed by a nonparametric approach [20]. To find a cut-point that maximizes the variable’s differentiating ability from the ROC curves the Youden index (*J*) was computed. *J* is defined as the variable’s value for which equal weight is given to sensitivity and specificity [21]. A two-tailed p value <0.05 was considered significant for all analyses. Data were analyzed using STATA version 14 (Stata Corp. College Station, TX, USA).

## Results

Descriptive baseline characteristics of patients are represented in Table 1. The whole population consisted of a relative low-moderate percentage of cases with type-II diabetes and CVD and a mild-moderate grade of kidney disease (median eGFR 54.1[30.0–84.6] ml/min/1.73m^2^). Conversely, proteinuria was severe, as shown by a median level above 1g/24h (1.5 [0.7–3.5]). Overall RI was 0.67±0.09. Primitive renal diagnoses were mainly represented by GN and TIN, being 55.6 and 31.8%, respectively, rather than by the group that included diabetic nephropathy DN, HTN and PKD which amounted to 12.7% in total. From the low (<0.65) to the high (>0.70) RI risk category, the frequency of diabetes, current smokers, CVD and levels of serum phosphorus were significantly increased. Conversely, eGFR, and haemoglobin levels showed a significant decreasing trend. Proteinuria levels were not different and use of calcium channel blockers was slight increased, even if not significantly, among RI risk categories.

**Table 1:** Basal characteristics of patients: Overall and by RI risk categories.

	Overall (n = 73)	<0.65 (n = 29)	0.65–0.70 (n = 15)	>0.70 (n = 29)	*p*
Age, *years*	57.8±17.0	54.9±16.6	56.3±17.7	61.5±16.9	0.318
Male gender, *%*	69.9	65.5	86.7	65.5	0.282
Diabetes, *%*	19.2	10.3	20.0	27.6	0.046
CVD, *%*	20.6	10.3	6.7	37.9	0.011
Smokers, *%*	30.1	17.2	40.0	37.9	0.038
Etiology of CKD, *%*					0.275
HTN/DN/PKD	12.7	7.7	7.1	21.7	
GN	55.6	61.5	71.4	39.1	
TIN	31.8	30.8	21.4	39.1	
BP, *mmHg*	132±16/77±10	131±13/78±8	126±18/76±10	136±17/77±11	0.138/0.795
Pulse Pressure, *mmHg*	54.4±13.0	53.1±11.5	49.9±13.1	58.7±13.9	0.105
PTH, *pg/mL*	191[57–364]	218[64–363]	137[26–226]	191[79–407]	0.545
eGFR, *mL/min/1.73 m*^*2*^	54.1[30.0–84.6]	69.6[51.0–96.6]	55.8[33.0–79.0]	30.0[16.5–59.7]	0.001
Urea, *mg/dL*	73.6±47.2	51.3±30.7	66.4±38.1	73.6±47.2	0.001
Phosphorus, *mg/dL*	3.7±0.7	3.5±0.6	3.6±0.7	4.0±0.7	0.015
Serum potassium, *mEq/L*	4.7±0.5	4.7±0.5	4.7±0.4	4.7±0.7	0.887
Uric acid, *mg/dL*	5.97±1.83	5.79±1.87	5.87±1.53	6.20±1.95	0.681
Hemoglobin, *g/dL*	13.0±2.2	14.1±2.0	12.9±2.3	12.0±1.9	0.001
Uprot, *g/24h*	1.5 [0.7–3.5]	1.2 [0.5–2.7]	1.3 [0.6–2.7]	3.0 [1.4–5.1]	0.085
Urinary Na, *mmol/24h*	137±70	131±83	146±55	138±64	0.835
RAAS-inhibitors, *% pts*	42.5	44.8	46.7	37.9	0.811
Ccb, *%pts*	28.8	13.8	33.3	41.4	0.062
Bb, *%pts*	19.2	13.8	20.0	24.1	0.604

CVD, cardiovascular disease; CKD, Chronic Kidney Disease; HTN, hypertensive nephropathy; DN, diabetic nephropathy; PKD, Polycystic kidney disease; GN, glomerulonephritis; TIN, tubulo-interstitial nephritis; BP, Blood pressure; PTH, Parathyroid Hormone; eGFR, estimated Glomerular Filtration Rate; RAAS, Renin-angiotensin-aldosterone-system; Ccb, Calcium channel blockers; Bb, Beta blockers; *p* value refers to *p* for trend between RI risk categories.

Continuous RI was significantly associated with age, serum phosphorus, eGFR, urea and haemoglobin. Mean RI levels were also higher in patients with a history of previous CVD, smoking habit and in the presence of diabetes (*p* = 0.001, 0.013, 0.033). Univariate associations between RI and overall proteinuria, systolic and diastolic blood pressure were not significant (*p* = 0.229, 0.184, 0.772 respectively). The univariate regression analyses are shown in Table 2.

**Table 2:** Univariate linear regression analysis for renal resistive index (RI) in CKD patients under nephrology care.

Characteristics	Coefficient (95% CI)	*p*
Age, *years*	0.0015 (0.0003–0.0027)	0.018
Gender *male (vs female)*	0.0067 (-0.0389–0.0523)	0.770
Diabetes *(yes vs no)*	0.0389 (0.0136–0.0912)	0.033
CVD, *%*	0.0870 (0.0394–0.1345)	0.001
Smokers *(yes vs no)*	0.0557 (0.0120–0.0995)	0.013
Etiology of CKD		
HTN/DN/PKD	1	
GN	-.0318(-0.0998–0.0363)	0.354
TIN	-.0055 (-0.0781–0.0672)	0.880
Systolic BP, *mmHg*	0.0009 (-0.0004–0.0023)	0.184
Diastolic BP, *mmHg*	-0.0003 (-0.0026–0.0019)	0.772
Pulse Pressure, *mmHg*	0.0016 (-0.0001–0.0032)	0.262
PTH, *pg/mL*	0.0001 (-0.0001–0.0001)	0.689
eGFR, for 10 *mL/min/1.73 m*^*2*^	-0.0141 (-0.0192- -0.0091)	<0.001
Urea, *mg/dL*	0.0011 (0.0008–0.0016)	<0.001
Phosphorus, *mg/dL*	0.0406 (0.0111–0.0701)	0.008
Serum potassium, *mEq/L*	0.0010 (-0.0410–0.0430)	0.973
Uric acid, *mg/dL*	0.0045 (-0.0070–0.0159)	0.442
Hemoglobin, *g/dL*	-0.0189 (-0.0277- -0.0102)	<0.001
Uprot, *g/24h*	0.0027 (-0.0018–0.0072)	0.229
Urinary Na, *mmol/24h*	-0.0001 (-0.0004–0.0002)	0.619
RAAS-inhibitors *(yes vs no)*	-0.0179 (-0.0601–0.0243)	0.400
Ccb *(yes vs no)*	0.0561 (-0.0917–0.1004)	0.154
Bb *(yes vs no)*	0.0442 (-0.008–0.0963)	0.196

CVD, cardiovascular disease; CKD, Chronic Kidney Disease; HTN, hypertensive nephropathy; DN, diabetic nephropathy; PKD, Polycystic kidney disease; GN, glomerulonephritis; TIN, tubulo-interstitial nephritis; BP, Blood pressure; PTH, Parathyroid Hormone; eGFR, estimated Glomerular Filtration Rate; RAAS, Renin-angiotensin-aldosterone-system; Ccb, Calcium channel blockers; Bb, Beta blockers.

From the univariate linear regression analysis, covariates with p<0.150 (age, diabetes, CVD, eGFR, Smoking habit, serum phosphorus, hemoglobin) were included in the multivariate linear regression model (Model 1, Table 3). Urea, although significant at univariate analysis, was not included in the Model 1 due to the collinearity with eGFR (VIF for Urea = 12.29). VIFs, after removing Urea from the model, were all <5, suggesting multicollinearity was not a concern. After backward selection of variables, with an elimination criterion of p<0.100, an history of previous CVD, the presence of type-II diabetes, smoking habit, lower eGFR and higher serum phosphate were significantly associated with higher RI levels. (Model 2, Table 3). These findings remained significant after adjusting for the main parameters that are known to be highly associated with RI such as age and hemoglobin, included in one or more steps of model building.

**Table 3:** Multivariate linear regression analysis for determinants of renal resistive index (RI).

Variables	Model 1		Model 2	
	coefficient (95% CI)	*p*	coefficient (95% CI)	*p*
Age (for 1 year)	0.001 (-0.001–0.002)	0.458	-	-
Diabetes, yes vs. no	0.026 (0.001–0.050)	0.048	0.024 (0.002–0.047)	0.042
CVD, yes vs. no	0.046 (0.003–0.089)	0.037	0.061 (0.015–0.106)	0.009
eGFR (for 10 mL/min)	-0.010 (-0.016- -0.003)	0.005	-0.011 (-0.016- -0.006)	<0.001
Smoking habit, yes vs. No	0.035 (0.009–0.072)	0.023	0.036 (0.007–0.074)	0.021
Serum phosphorus, (for 1 mg/dL)	0.048 (0.020–0.076)	0.001	0.047 (0.021–0.073)	0.001
Hemoglobin, (for 1 g/dL)	-0.004 (-0.013–0.005)	0.334	-	-

CI, Confidence Intervals; CVD, cardiovascular disease; eGFR, estimated Glomerular Filtration Rate.

**Fig 1:**
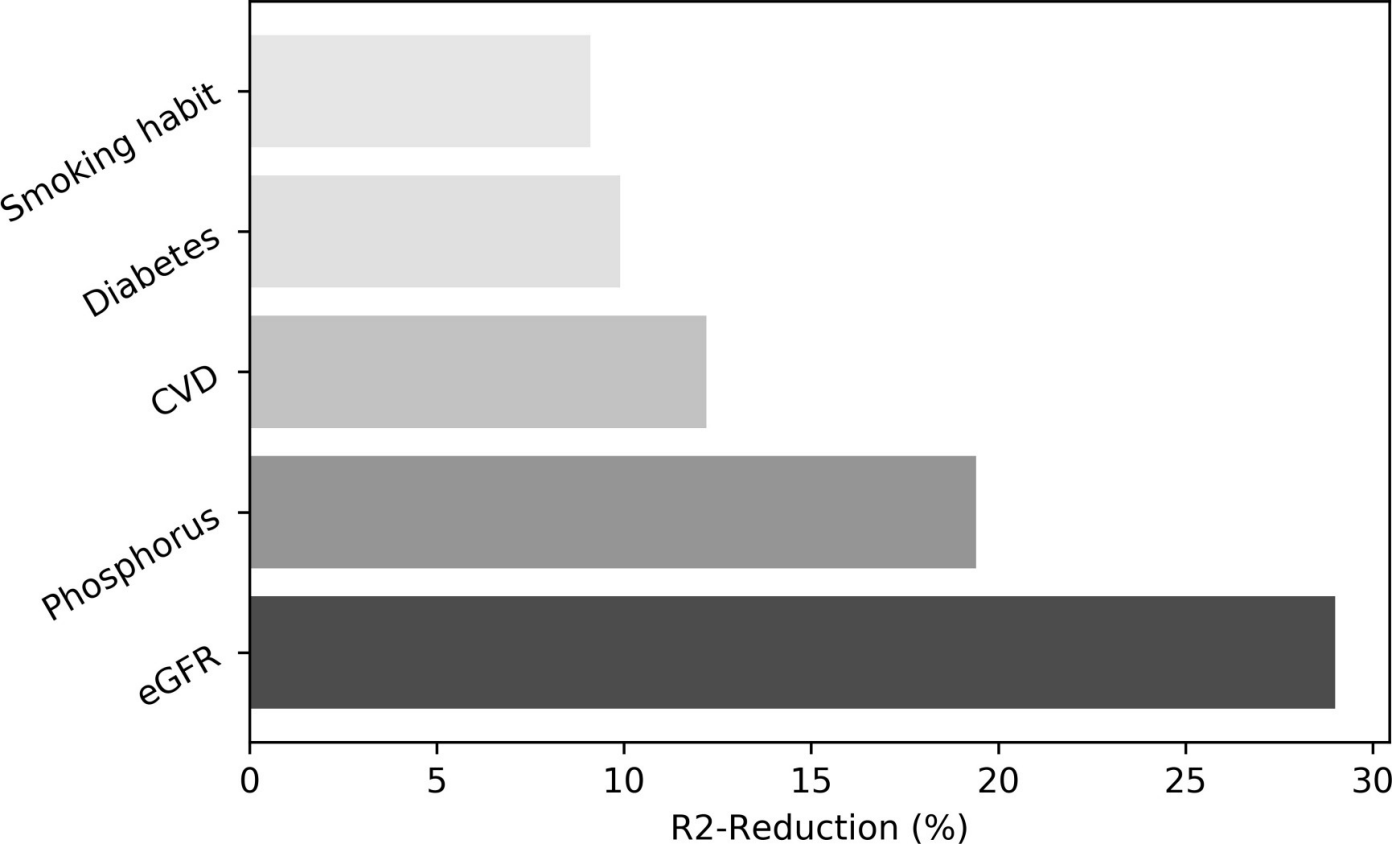
Individual contribution (expressed as %) of covariates included in Model 2 to the overall model fit according to R^2^ reduction analysis. Grey tones are graduated based on the amount of contribution of each variable. eGFR, estimated Glomerular Filtration Rate; CVD, cardiovascular disease.

According to R^2^ reduction analysis, the individual contribution to the variance of the final model (Model 2) was consistent for all significant predictors and was slightly predominant for eGFR (29.0%), serum phosphorus (19.4%) and CVD (12.2%) as compared to diabetes (9.9%) or smoking habit (9.1%) (Fig 1). No significant interaction effect between covariates included in the Model 2 was found.

**Fig 2:**
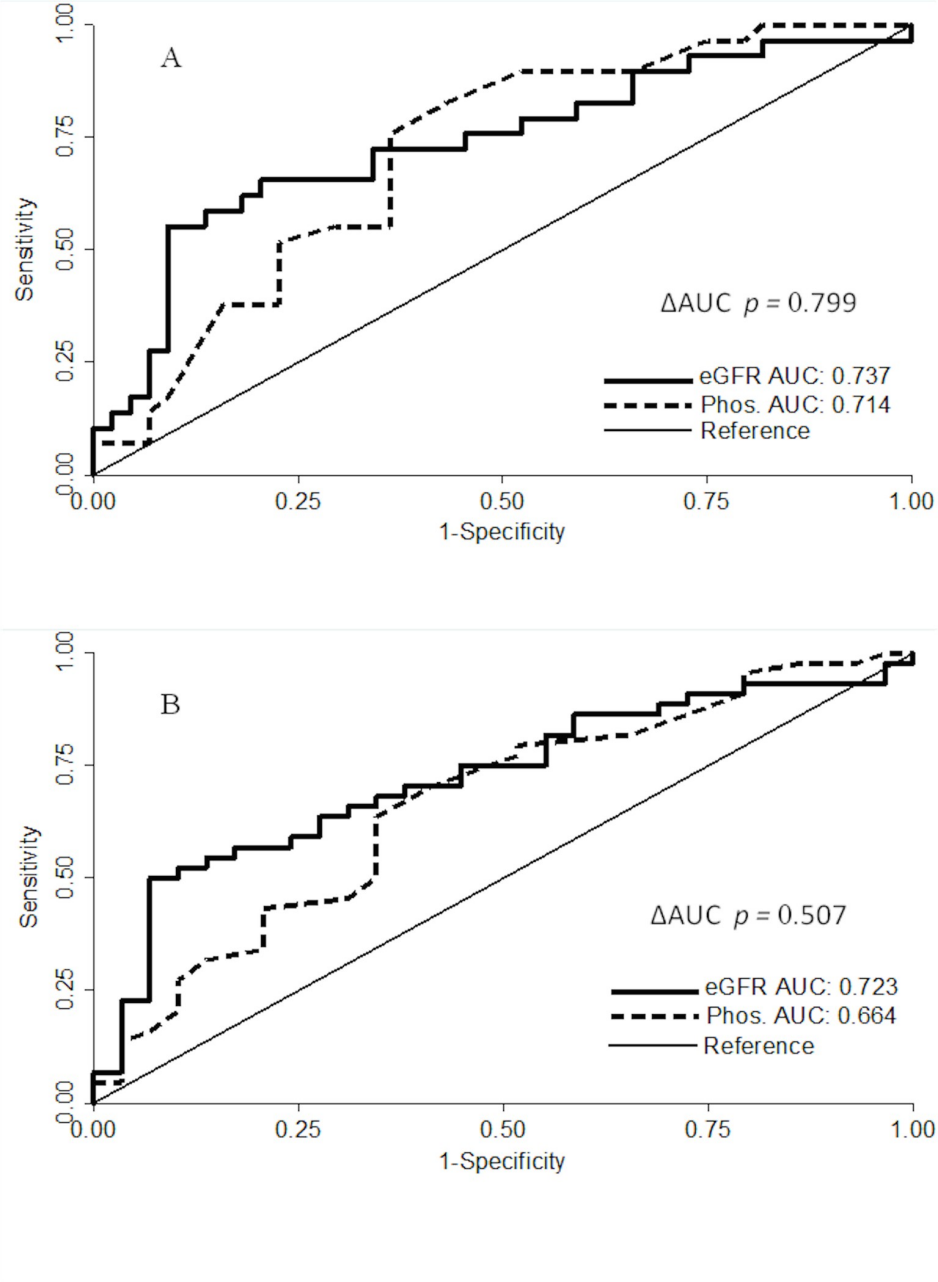
Receiver operating characteristic curves (ROC) for the combination of serum phosphorus levels and eGFR values on high RI levels (reference: RI > 0.70, panel A; RI ≥ 0.65 panel B). eGFR, estimated glomerular filtration rate; Phos., serum phosphorus, AUC, Area under the curve.

ROC curves (Fig 2) showed that serum phosphorus has a discriminatory power almost equal to that of eGFR in identifying a high RI level. Values of Area Under the Curves (AUC) for eGFR and serum phosphorus when considering the cut-off of RI ≥0.65 (Fig 1, Panel B) or the RI >0.70 (Fig 1, Panel A), were similar and did not reach statistical difference (*p* = 0.507 and *p* = 0.799, respectively). When the cut-points *J* were derived from the ROC curves, the two points, 4.0 (Sensitivity = 71.5%, Specificity = 63.6%, Odds Ratio = 3.32: 95% CI 1.24–8.87) and 4.3 (Sensitivity = 82.8%, Specificity = 65.6%, Odds Ratio = 6.32: 95% CI 2.03–19.61) mg/dL of serum phosphorus, were able to identify patients with RI ≥0.65 and >0.70, respectively.

## Discussion

US-Doppler imaging has already been defined as a reliable tool for assessing the severity of CKD. The advantages of this method are represented by its ability to detect macroscopic vascular abnormalities in the kidney and to provide important diagnostic and prognostic information [22]. Moreover, the increasing use of RI as a predictor of bad outcomes in CKD patients, such as the eGFR decline, encourages a more detailed investigation of the clinical parameters that may be associated with a worsening of US metrics. For the first time, we specifically explored the clinical profile of patients undergoing US Doppler evaluation in a cohort of patients referred to nephrologists, the so-called tertiary care setting. These patients represent a selected population with peculiar clinical characteristics with respect to unreferred patients, including younger age, more advanced disease, higher burden of cardiovascular comorbidities, and higher BP [23–25]. Thus, the results obtained from this cohort are not directly generalizable.

The first main finding of our study is that a combination of modifiable and non-modifiable risk factors, regardless of eGFR levels, is able to predict higher RI. In particular, mean RI, measured at baseline study visit, was about 0.024 (p = 0.042) and 0.061 (p = 0.009) higher in patients with type II diabetes or a positive history for CVD, 0.036 (p = 0.021) higher in smokers as compared to non-smokers. When effect of continuous variables was tested, RI was on average 0.047 higher for each unit increase in serum phosphate levels (p = 0.001) and 0.011 lower for each 10 mL/min/1.73m^2^ of eGFR increase (p<0.001). The contribution of each significant risk factor to the overall model fit was in agreement with the previous result, being the eGFR, serum phosphorus levels and previous CVD the major risk factors which may explain a change in RI category, with an additive and independent effect (No interaction effect has been observed). The second important finding is that clinical correlates of RI in a tertiary nephrology care setting should be supplementary to those found in the general population [11,22].

Several previous studies have examined the factors associated with a higher ultrasound RI. Ponte et al. found that systolic and diastolic blood pressure, age and heart rate were significantly associated with a worsening in RI in the general population [26]. However, the different setting of enrolled patients makes it hard to perform a valid comparison with our data. This is also confirmed by the finding that, in the analysis carried out by Ponte and Colleagues, renal function appeared to be not related to RI, which is controversial when compared with data obtained from other studies [10,11,16]. Several previous studies have reported a strict relationship between RI and blood pressure, being RI directly related to systolic blood pressure and inversely associated to mean arterial pressure and diastolic blood pressure [27]. These findings have been confirmed in patients selected from general population, in critically ill patients admitted to intensive care units, as well as in those with CKD [28–30]. In our cohort, interestingly we found no association between systolic, diastolic or pulse pressure values with RI in either univariate or multivariate analysis. One possible explanation is that patients included in our study show a mild reduction of eGFR (median 54.1 mL/min/1.73m^2^). In the analysis by Doi et al. who included patients with similar degree of kidney dysfunction, they report no significant difference of systolic and diastolic blood pressure between RI levels above and below the median. A significant trend of blood pressure parameters between RI categories was evident only when Authors stratified analysis by eGFR level (≥ and < 60 mL/min/1.73m^2^) [11]. Thus, we could not exclude that a more advanced degree of kidney damage is needed to observe this correlation in the specific setting of CKD patients, as showed elsewhere [30]. Another finding of our study is that age has been found a non significant determinant of RI at adjusted analysis. The link between ageing and RI is definitely an old concept. Indeed, it has been demonstrated that RI increases with ageing, even in healthy subjects [31]. Higher RI values were found in older patients as compared to those with a younger age, these results being confirmed in many clinical setting including CKD patients [11,30]. On the other hand, the effect of age seems to be the final product of a clinical interaction with other extrarenal factors such as stiffness of prerenal vessels or cardiovascular comorbidities [32]. Thus, it is possible that after adjusting for several CV risk factors (i.e. diabetes, CVD and smoking habit) the effect of age is no longer significant. Conversely, we could not exclude that the small sample of our cohort influenced this finding. Our study shows also some methodological differences from previous studies. Almost all studies modeled univariate associations between eGFR and RI rather than testing whether the association of eGFR with RI is independent from other covariates as well as whether the association of some other predictors with RI is independent from eGFR. Determinants of RI were also described for early renal transplant recipients, but rarely for patients with renal dysfunction referred to nephrologists [33]. Indeed, the only study that was conducted in a cohort of tertiary nephrology care outpatients, depicted the prognostic role of RI on renal outcome, but the authors did not search for its clinical correlates [10]. A controversial issue of these previous analyses was the level of RI to be considered above which the risk increases. Some studies reported the risk estimates plotted around the tertiles or median RI values as cut-offs [11,33,34], whereas others studies considered 0.65 or 0.70 as correct thresholds to define risk [10,16]. This biphasic risk classification of RI is supported by the demonstrations that an RI value of 0.65 is able to detect several patterns of kidney damage, such as tubular and glomerular injury [15]. On the other hand, an RI of 0.70 is considered the lower point that allows to predict a decline in eGFR over time [10,16]. In our study, we evaluated the determinants of RI as continuous variable, thus avoiding influencing the results based on the variable distribution within our cohort.

The association between RI and type II diabetes has been already described by Matsumoto et al. who found increased RI levels in patients with renal dysfunction secondary to diabetes compared with non-diabetic CKD patients [35]. The possible underlying mechanism is that in diabetic patients, multivessel disease based on systemic atherosclerotic disorders could affect renal injury through long-term intrarenal ischemia. A similar pattern may depend upon the link between a history of CVD and the worsening in RI as evidenced by our results. The previous CVD events that we measured are, indeed, principally caused by atherosclerosis which is a potent trigger of renal dysfunction [36,37]. Another possible hypothesis that may explain the link between CVD and renovascular damage is an up-regulation of the renin-angiotensin-aldosterone-system (RAAS) that is a major target of several drugs used to relent CKD progression. Moreover, it has already been demonstrated that the presence of each CVD event contributes to a further impairment in RI [38]. On the other hand, the lack of association with the presence of proteinuria can be explained by the fact that RI is not generally correlated with proteinuria in chronic glomerulonephritis, which is the main cause of renal diagnosis in our patients, because albuminuria in patients with chronic glomerulonephritis may be more likely derived from glomerular capillary damage alone without (or with less) damage to the main renal artery [39]. This latter concept is in agreement with the recent finding, reported by Rozemeijer et al. in a cohort of adult critically ill patients, that RI is not directly determined by markers of blood flow in microcirculation [28]. Moreover, while the presence of proteinuria is a well-defined risk factor of glomerular and tubular damage and kidney disease progression, it is also considered a marker of endothelial dysfunction [40]. Hence, further studies are needed to clarify the controversial role of other determinants of RI, such as parameters of microcirculation.

Our study could be considered a hypothesis-generating study, with the main findings being represented by the relationships between serum phosphorus and renal RI. Even if a causal-effect mechanism requires further studies to be elucidated, we can suppose that the significant association of RI with higher serum phosphate can be attributable to the fact that an increase in serum phosphate across the kidney may cause vascular damage by either the direct effect of phosphate or formation of calcium-phosphate crystals [33,41,42]. Vascular-endothelial dysfunction has been previously studied in more detail. Indeed, increased serum phosphate levels is responsible for different types of vascular disease, such as intima-media thickness, vascular stiffness and peripheral vascular disease that may explain, at least in part, the change of RI in the same direction [43,44]. Nevertheless, since the effect of serum phosphate on RI, in our multivariable models, was significant, even after adjustment for the CVD assessment, which involves systemic atherosclerotic disease, some other mechanisms seem to be implicated. Besides the vascular-endothelial dysfunction, the increase of serum phosphate levels is directly responsible for tubular injury and interstitial fibrosis [41,42]. Both these factors determine an increase in interstitial pressure across the kidney which is, together with the vascular compliance, a major determinant of RI [45]. Beyond the exact pathways of damage, it is remarkable that serum phosphate plays an important prognostic role specifically in CKD patients. In the past decades, several studies have shown a strong correlation between higher serum phosphate levels and the increased risk of CKD progression, cardiovascular events and mortality [46–48]. Other than in a non-dialysis setting, an increased mortality risk in patients with higher serum phosphate levels was found in patients who start dialysis [49]. Although our study was performed in patients treated with a conservative predialytic approach, it would not be surprising if the same association was demonstrated in dialysis patients. Indeed, we found that the association between RI and serum phosphate is independent from renal function. With respect to smoking habit, several prospective studies have demonstrated that cigarette smoking is strictly associated with a higher CKD progression rate in different clinical conditions, such as diabetic nephropathy, IgA nephropathy and membranous idiopathic nephropathy [50–52]. Various mechanisms have been indicated to explain the nephrotoxic effect of smoking. First of all, smoking has chronic effects, by diminishing nitric oxide availability and endothelial cell–dependent vasodilation, which lead to an enhanced oxidative stress, glomerulosclerosis and tubular atrophy [53,54]. Moreover, cigarette smoke contains glycotoxins which induce advance glycation end products (AGEPs) and thus directly promote pathological vascular changes [55]. On the other hand, evidence of a link between smoking habit and Doppler-RI impairment are limited. The current smokers selected from a cohort of hypertensive patients and from a cohort of patients suffering from chronic obstructive pulmonary disease (COPD), have simultaneously higher RI levels, but the independent association of smoking habit as a risk factor of increasing RI has not been shown [11, 56]. However, based on our data, we cannot exclude that the measures of duration of smoking and number of cigarette/per day could explain, at least in part, this unfavorable pattern.

Our study has some limitations. Firstly, the largest part of renal diagnosis of our cohort was attributable to GN and TIN, thus not completely representative of overall cause of CKD on the large scale. Secondly, the single-center dimension of this study limits the generalizability of results. Thirdly, only few patients were treated with phosphate binders, thus excluding the possibility of testing if the link between phosphate and RI would be modified by their use in therapy. Fourthly, the cross-section study design has the intrinsic limitations to measure all variables in a single time-point. At the same time, a remarkable advantage of this design is to allow investigators to consider many putative risk factors at the same time. For this reason, it has been recently encouraged for the implementation of cross-sectional design in clinical research [57]. These limitations, together with the used sample size, indicate the preliminary nature of these interesting results.

In conclusion, in CKD patients followed in the setting of an outpatient renal clinic, the clinical profile of patients with higher RI levels includes those with low eGFR levels, diabetes, previous CVD, higher serum phosphate and smoking habit. Further studies are needed to verify whether high RI indicates a more complex pathway of intrarenal damage, which is, besides and beyond the eGFR value and systemic atherosclerosis. Results also call for more intervention studies, i.e. randomized clinical trials, testing the effect of drugs that, by modifying parameters associated with RI, could improve cardio-renal prognosis in CKD patients.

## Supporting information

S1 File (SAV)

## Abbreviations

CKD: chronic kidney disease
eGFR: estimated glomerular filtration rate
RI: renal resistive index
US: ultrasonography
CVD: previous cardiovascular disease
DN: diabetic nephropathy
HTN: hypertensive nephropathy
GN: glomerulonephritis
TIN: tubulo-intersitial nephritis
PKD: polycystic kidney disease
SD: standard deviation
IQR: interquartile range
AIC: Akaike information Criterion
ROC: receiver operating characteristic
J: Youden index
AUC: Area Under the Curves
RAAS: renin-angiotensin-aldosterone-system
AGEPs: advance glycation end products
COPD: chronic obstructive pulmonary disease
BP: Blood pressure
PTH: Parathyroid Hormone
Ccb: Calcium channel blockers
Bb: Beta blockers
OR: Odds Ratio
CI: Confidence Intervals
